# Age estimation of individuals aged 5–23 years based on dental development of the Indonesian population

**DOI:** 10.1080/20961790.2021.1886648

**Published:** 2021-04-15

**Authors:** Adisty Setyari Putri, Nurtami Soedarsono, Benindra Nehemia, Djaja Surya Atmadja, Douglas H. Ubelaker

**Affiliations:** a Department of Oral Biology, Division of Forensic Odontology, Faculty of Dentistry, Universitas Indonesia, Jakarta, Indonesia; b Department of Dento-Maxillofacial Radiology, Faculty of Dentistry, Universitas Indonesia, Jakarta, Indonesia; c Department of Forensic Medicine and Medico-legal, Faculty of Medicine, Universitas Indonesia, Jakarta, Indonesia; d Department of Anthropology, National Museum of Natural History, Smithsonian Institution, Washington, DC, USA

**Keywords:** Forensic sciences, forensic odontology, age estimation, tooth calcification, tooth eruption, root resorption

## Abstract

Dental development can be used to estimate age for forensic purposes. However, most of the currently available methods are less reliable for the Indonesian population due to population variability. This study presents a new method and evaluates other methods that utilize dental development to estimate the age of Indonesian people. Panoramic radiographs of 304 young Indonesian people aged 5–23 years old were analysed for deciduous tooth root resorption, permanent tooth calcification, and eruption. The extent of tooth root resorption was determined based on AlQahtani’s modified Moorrees et al. method. Tooth calcification was classified based on a modified Demirjian et al. method. Tooth eruption was evaluated based on AlQahtani’s modified Bengston system. The sequence of tooth root resorption, and permanent tooth calcification and eruption were grouped into 19 age categories (from 5–23 years old) in an atlas. The differences between males and females, between maxillary and mandibular teeth, and between right and left teeth were also analysed. There were minimal significant differences of tooth development between males and females, and between the right and left teeth (*P* > 0.05), while the maxillary and mandibular dental development was significantly different (*P* < 0.05). The newly developed atlas showed the development of the right side of maxillary and mandibular tooth of combined sex of Indonesian population. Another 34 panoramic radiographs of known-age and sex individuals from Indonesia were assessed using the newly developed Atlas of Dental Development in the Indonesian Population, Ubelaker’s Dental Development Chart, The London Atlas of Human Tooth Development and Eruption by AlQahtani, and the Age Estimation Guide-Modern Australia population by Blenkin-Taylor. Accuracy was assessed by comparing estimated age to actual chronological age using the Bland-Altmand test. Results show that the smallest range of error was found in the Atlas of Dental Development in the Indonesian Population (−0.969 to 1.210 years), followed by The London Atlas of Human Tooth Development and Eruption by AlQahtani (−2.013 to 1.990 years), the Age Estimation Guide-Modern Australia population by Blenkin-Taylor (−2.495 to 2.598 years), and the Dental Development Chart by Ubelaker (−2.960 to 3.289 years). These findings show that the Atlas of Dental Development constructed in this study performs better than the other three methods and presents greater accuracy of age estimation in the Indonesian population.

**Key points**
- Dental development such as deciduous tooth root resorption, permanent tooth calcification, and tooth eruption can be used to estimate age for forensic purposes.
- The development of the teeth are influenced by genetic, ethnicity, and sex, therefore an age estimation method must be constructed based on the same population.
- There were minimal significant differences in tooth development between male and female, and between right and left teeth, but there was significant difference between maxillary and mandibular teeth.
- The Atlas of Dental Development in the Indonesian Population constructed in this study allowed more accurate age estimation of the Indonesian sample than the other methods tested.

Supplemental data for this article are available online at https://doi.org/10.1080/20961790.2021.1886648.

## Introduction

Age estimation provides important information in personal identification of unidentified persons in mass disaster and age forgery cases [1–3]. In Indonesian Criminal Law, the age of an individual is crucial, because the acts, the articles and the punishment vary with the age. Errors in estimation of the age can result in errors in legal procedures and punishment [4].

Teeth have been widely used to estimate the age of individual [5]. The development of teeth, as well as degenerative changes, occurs at a certain age, therefore it can be used to estimate age of an individual from intra-uterine period to adulthood [6,7]. Age estimation by dental development using radiographic methods include the scoring procedure by Demirjian et al. [8], and the atlas methods by Schour and Massler [9], Ubelaker [10], AlQahtani et al. [11], and Blenkin and Taylor [12]. All of these methods were developed based on studies of non-Indonesian samples. Dental development are influenced by genetics, ethnicity, and sex, but less influenced by socio-economic and environmental factors [13]. Due to ethnicity influence, differences on the timing and rate of dental development can occur among different populations [3]. Since the Indonesian population consists of many ethnicities, a specific dental development population dataset must be assessed to get the accurate estimation of age of the Indonesian.

This study examines the stages of deciduous tooth root resorption, permanent tooth calcification and permanent tooth eruption through radiographic assessment to evaluate how dental development can be employed to estimate age in Indonesian people. Another aim is to compare the accuracy of ages estimated using the new Indonesian Atlas and three other systems: the Dental Development Chart by Ubelaker (1978), The London Atlas of Human Tooth Development and Eruption by AlQahtani (2010), and the Age Estimation Guide-Modern Australia population by Blenkin-Taylor (2012) when applied to a sample of the Indonesian population of known age.

## Materials and methods

This cross-sectional, and retrospective study utilized panoramic radiographs of patients of the Dental Hospital Faculty of Dentistry, Universitas Indonesia from 2010–2014, aged 5–23 years with written informed consents signed by the patients or their guardians. Patients included in the study met the following criteria: all teeth were clearly visible on panoramic radiographs, no variation in the teeth number, no impaction, no abnormal persistence of deciduous tooth, no extracted or premature loss of deciduous teeth, no deciduous tooth root resorption caused by factors other than its permanent successor, no periapical abnormalities, no tooth abnormalities, and no fixed orthodontics appliances.

The study utilized 304 panoramic radiographs, grouped into 19 age categories (5–23 years), each including 16 samples. Each age group consisted of 8 samples of males and 8 samples of females (Table 1).

**Table 1.** Sample distribution of age group and sex of 304 panoramic radiograph.

Age group (years)	Sex		Sum
	Male	Female	
5 (4.51–5.50)	8	8	16
6 (5.51–6.50)	8	8	16
7 (6.51–7.50)	8	8	16
8 (7.51–8.50)	8	8	16
9 (8.51–9.50)	8	8	16
10 (9.51–10.50)	8	8	16
11 (10.51–11.50)	8	8	16
12 (11.51–12.50)	8	8	16
13 (12.51–13.50)	8	8	16
14 (13.51–14.50)	8	8	16
15 (14.51–15.50)	8	8	16
16 (15.51–16.50)	8	8	16
17 (16.51–17.50)	8	8	16
18 (17.51–18.50)	8	8	16
19 (18.51–19.50)	8	8	16
20 (19.51–20.50)	8	8	16
21 (20.51–21.50)	8	8	16
22 (21.51–22.50)	8	8	16
23 (22.51–23.50)	8	8	16
Total	152	152	304

**Figure 1.**
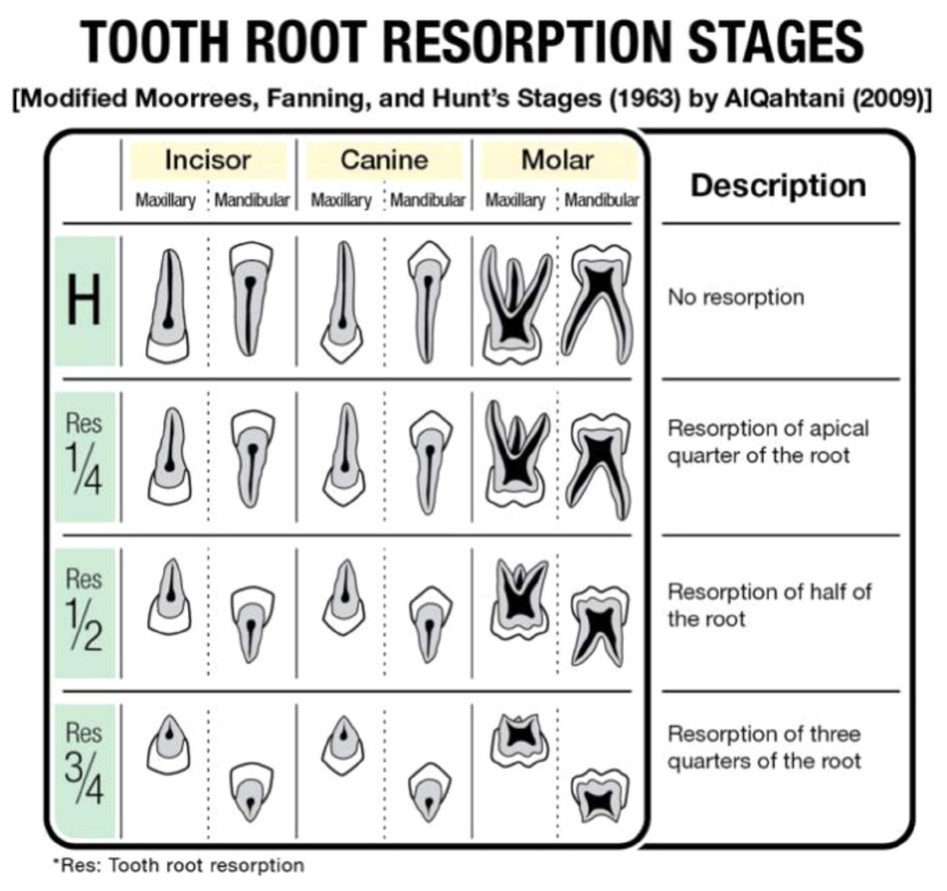
Description of tooth root resorption stages.

The parameters analysed in this study were the stages of deciduous tooth root resorption, permanent tooth calcification, and permanent tooth eruption of each tooth in the sample. The deciduous tooth root resorption stage was assessed according to the method of Moorrees et al. (1963), which was modified by AlQahtani (Figure 1). The four stages of deciduous tooth root resorption for a single or double root are H (roots intact), Res ¼ (one-fourth of apical root resorption), Res ½ (half apical root resorption), and Res ¾ (three-fourth of apical root resorption) [11].

**Figure 2.**
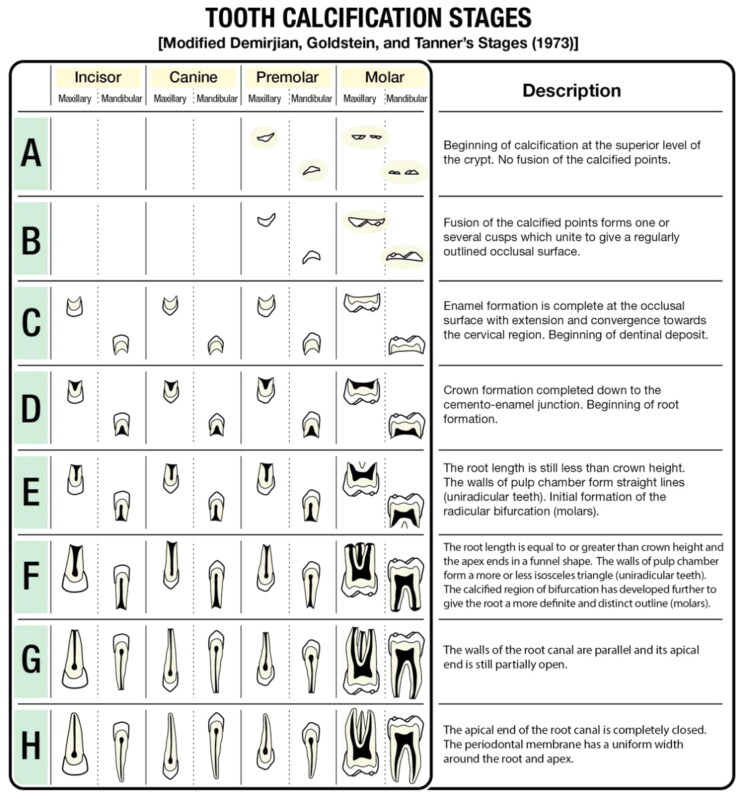
Description of tooth calcification stages.

The permanent tooth calcification stage was assessed according to the modified Demirjian calcification stage (Figure 2). There are eight stages of tooth calcification between stages A and H for molars and premolars, and six stages of tooth calcification (stage C to H) for incisors and canines. Stage A is determined when the crypt begins to calcify at the superior level and stage H is determined when the apical area of the root is completely closed [9,14].

**Figure 3.**
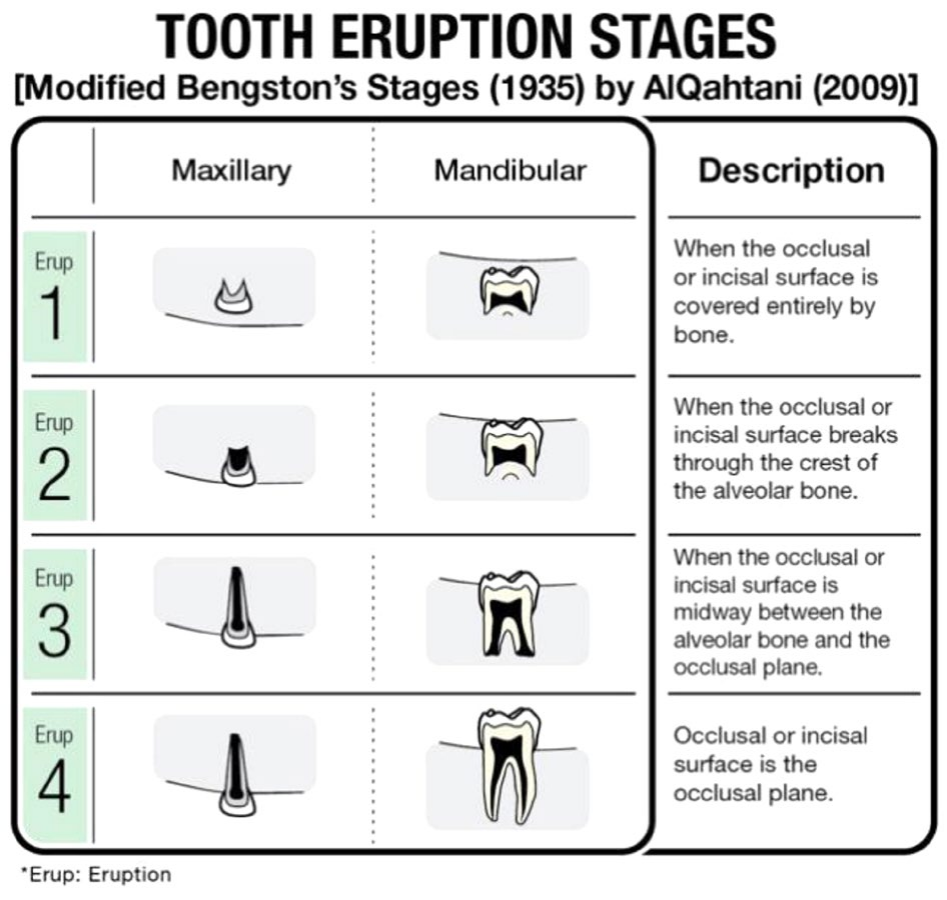
Description of tooth eruption stages.

Permanent tooth eruption was classified according to the method of Bengston, which was modified by AlQahtani (Figure 3). There are four stages of tooth eruption ranging from Eruption 1, when the bone is covering the occlusal or incisal surface completely, to Eruption 4, when the incisal or occlusal surface reaches the occlusal plane [11] (Figures 1–3).

Statistical analysis was performed using SPSS version 20 (IBM Corp., Armonk, NY, USA) for univariate analysis, comparison test, and the Kappa test. The Atlas of Dental Development in the Indonesian Population was constructed based on the modal stage of each deciduous tooth root resorption, each permanent tooth calcification and each permanent tooth eruption in every age group between 5 and 23 years and among both sexes. The minimum and maximum stages were also analysed for each tooth in each age group. The differences between male and female dental development were analysed using Chi-square test 2 × 2 or Kolmogorov Smirnov test 2 × K, while the differences between maxillary and mandibular teeth, and between right and left teeth were analysed using McNemar test for 2 × 2 table or Wilcoxon test for 2×(>2) tables.

Another 34 panoramic radiographs of known-age and sex individuals from Indonesia were assessed using the Atlas of Dental Development in the Indonesian Population, the Dental Development Chart by Ubelaker, The London Atlas of Human Tooth Development and Eruption by AlQahtani, and the Age Estimation Guide-Modern Australia population by Blenkin-Taylor to find the most accurate estimated age compared to the known chronological age using the Bland-Altmand test and the limit was set to ±2 years (Table 2).

**Table 2.** Sample distribution of age and sex of 34 panoramic radiograph.

Age group (years)	Sex		Sum
	Male	Female	
5 (4.51–5.50)	2	0	2
6 (5.51–6.50)	2	0	2
7 (6.51–7.50)	0	2	2
8 (7.51–8.50)	1	2	3
9 (8.51–9.50)	2	0	2
10 (9.51–10.50)	1	1	2
11 (10.51–11.50)	0	2	2
12 (11.51–12.50)	1	1	2
13 (12.51–13.50)	1	0	1
14 (13.51–14.50)	1	0	1
15 (14.51–15.50)	2	0	2
16 (15.51–16.50)	0	1	1
17 (16.51–17.50)	0	2	2
18 (17.51–18.50)	1	1	2
19 (18.51–19.50)	0	1	1
20 (19.51–20.50)	1	1	2
21 (20.51–21.50)	1	0	1
22 (21.51–22.50)	0	2	2
23 (22.51–23.50)	1	1	2
Total	17	17	34

The inter-observer and intra-observer reliability test to determine the stages of tooth root resorption, calcification, and eruption was performed on 37 random subject from the 304 panoramic samples; the test to determine estimated age based on the Atlas of Dental Development in the Indonesian Population and the three other methods was performed on 10 random subjects from the 34 panoramic samples. The inter-observer reliability test was carried out by two authors (ASP and BN) on the same samples, while the intra-observer reliability test was conducted by the first author (ASP) twice for the same samples at 2 weeks interval from the initial measurement.

## Results

Intra-observer and inter-observer test result shows excellent agreement. The Kappa value to determine the stages of tooth root resorption, calcification, and eruption was 0.95 for the inter-observer test and 0.978 for the intra-observer test, while the Kappa value to determine estimated age based on the Atlas of Dental Development in the Indonesian Population and the three other methods was 0.836 for the inter-observer test and 0.917 for the intra-observer test.

The differences in dental development between females and males were analysed statistically and showed no significant differences (*P* > 0.05) for all teeth of the three parameters in all age group, except for root resorption of tooth 64 (maxillary left first deciduous molar) in the 6-year old age group that was significantly different (*P* < 0.05). Based on the findings, the modal, minimum, and maximum stage of each deciduous tooth root resorption, permanent tooth calcification and eruption in every age group between 5 and 23 years were combined between male and female, and can be used for both sexes (Supplementary Table S1).

**Figure 4.**
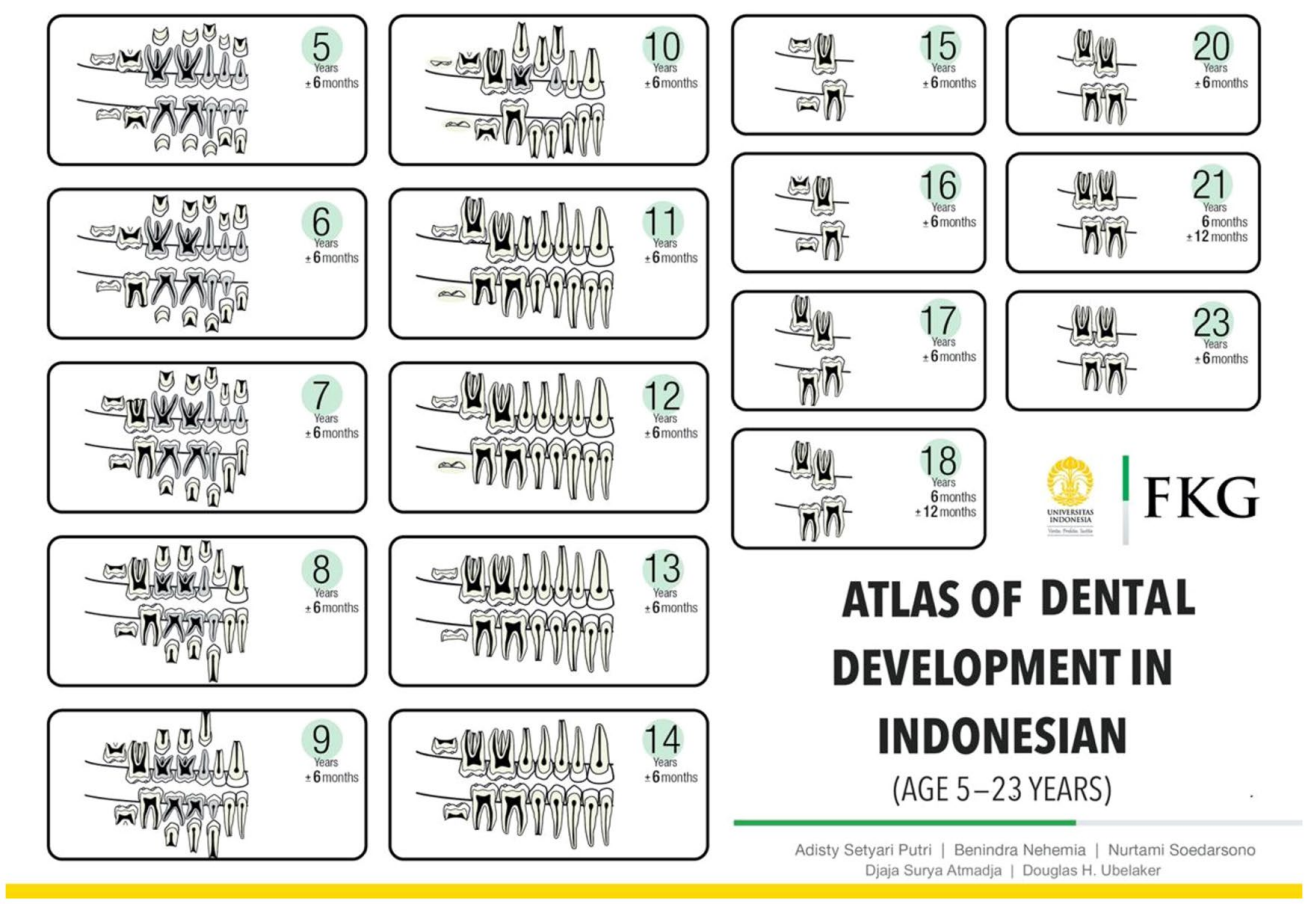
Atlas of Dental Development in the Indonesian Population (age 5–23 years).

Determination of the dental development differences between the right and left teeth, and between the maxillary and mandibular teeth of each age group was performed. The results revealed no differences between the right and left deciduous tooth root resorption, permanent tooth calcification and permanent tooth eruption in each age group of 5–23 years. Whereas, the analysis between the maxillary and mandibular teeth indicated significant differences between dental development of the maxillary and the mandibular teeth (Table 3–5. Incorporating these results, the Atlas of Dental Development illustrates the right region of the maxillary and mandibular teeth. The atlas was created using Adobe Illustrator CS6 and the publication Dental Embryology, Histology, and Anatomy (Bath-Balogh, 2006) [15] (Figure 4).

**Table 3.** *P*-value analysis of deciduous root resorption between the maxillary and mandibular teeth.

Age group (years)	Tooth 85–Tooth 55	Tooth 84–Tooth 54	Tooth 83–Tooth 53	Tooth 82–Tooth 52	Tooth 81–Tooth 51
5	0.317	0.014*	1.000	0.035*	0.034*
6	0.500	0.031*	1.000	0.417	0.372
7	1.000	0.014*	0.014	0.066	0.003*
8	0.705	0.248	0.010*	0.009*	0.180
9	0.025*	0.317	0.029*	0.102	1.000
10	0.679	0.392	0.061	1.000	1.000
11	0.276	1.000	0.317	1.000	1.000
12	0.157	1.000	0.102	1.000	1.000

* *P* < 0.05.

**Table 4.** *P*-value analysis of tooth calcification between the maxillary and mandibular teeth.

Age group (years)	Tooth 48–Tooth 18	Tooth 47–Tooth 17	Tooth 46–Tooth 16	Tooth 45–Tooth 15	Tooth 44–Tooth 14	Tooth 43–Tooth 13	Tooth 42–Tooth 12	Tooth 41–Tooth 11
5	1.000	1.000	0.157	0.549	0.250	0.705	0.011*	0.002*
6	0.317	0.564	0.082	0.705	0.082	1.000	0.014*	0.011*
7	1.000	1.000	0.223	1.000	0.020*	0.206	0.002*	0.007*
8	1.000	0.564	1.000	1.000	0.046*	0.135	0.000*	0.001*
9	0.157	0.500	1.000	0.317	0.083	1.000	0.001*	0.001*
10	0.083	0.317	1.000	0.368	0.083	0.157	0.002*	0.002*
11	0.082	0.368	1.000	0.250	1.000	0.500	0.180	0.500
12	0.414	0.135	0.317	0.564	0.317	0.135	0.096	0.083
13	0.506	0.083	0.317	0.014*	1.000	0.025*	0.157	0.157
14	0.135	1.000	1.000	0.500	0.500	0.031*	0.083	0.317
15	0.223	1.000	1.000	1.000	1.000	0.063	0.317	1.000
16	0.317	1.000	0.317	0.317	0.317	1.000	1.000	1.000
17	0.564	1.000	1.000	1.000	1.000	1.000	1.000	1.000
18	0.223	0.157	1.000	0.317	1.000	1.000	0.317	1.000
19	0.368	1.000	1.000	1.000	1.000	1.000	1.000	1.000
20	0.083	0.317	1.000	1.000	1.000	1.000	1.000	1.000
21	0.180	0.157	1.000	1.000	1.000	1.000	1.000	1.000
22	0.083	0.317	1.000	1.000	1.000	1.000	1.000	1.000
23	0.008*	1.000	1.000	1.000	1.000	1.000	1.000	1.000

* *P* < 0.05.

**Table 5.** *P*-value analysis of tooth eruption between the maxillary and mandibular teeth.

Age group (years)	Tooth 48–Tooth 18	Tooth 47–Tooth 17	Tooth 46–Tooth 16	Tooth 45–Tooth 15	Tooth 44–Tooth 14	Tooth 43–Tooth 13	Tooth 42–Tooth 12	Tooth 41–Tooth 11
5	1.000	1.000	0.317	1.000	1.000	1.000	0.317	0.157
6	0.317	1.000	0.102	1.000	1.000	1.000	0.180	0.024*
7	1.000	1.000	0.020*	1.000	1.000	1.000	0.020*	0.005*
8	1.000	0.317	0.157	1.000	0.414	1.000	0.038*	0.066
9	1.000	1.000	1.000	1.000	0.317	0.157	0.156	1.000
10	1.000	0.096	1.000	0.083	0.458	0.008*	0.414	0.157
11	1.000	0.096	1.000	0.785	0.157	0.317	1.000	1.000
12	1.000	0.082	1.000	0.109	0.083	0.066	0.157	1.000
13	1.000	0.180	1.000	0.083	1.000	0.157	0.317	1.000
14	0.083	0.500	1.000	1.000	1.000	0.317	1.000	1.000
15	0.180	1.000	1.000	1.000	1.000	0.157	1.000	1.000
16	0.014*	1.000	1.000	1.000	1.000	1.000	1.000	1.000
17	0.083	1.000	1.000	1.000	1.000	1.000	1.000	1.000
18	0.019*	1.000	1.000	1.000	1.000	1.000	1.000	1.000
19	0.705	1.000	1.000	1.000	1.000	1.000	1.000	1.000
20	0.368	1.000	1.000	1.000	1.000	1.000	1.000	1.000
21	1.000	1.000	1.000	1.000	1.000	1.000	1.000	1.000
22	0.250	1.000	1.000	1.000	1.000	1.000	1.000	1.000
23	1.000	1.000	1.000	1.000	1.000	1.000	1.000	1.000

* *P* < 0.05.

The comparative accuracy of estimating age using four dental development methods (The Atlas of Dental Development in the Indonesian Population, the dental development chart by Ubelaker (1978), The London Atlas of Human Tooth Development and Eruption by AlQahtani (2010), and the Age Estimation Guide-Modern Australia population by Blenkin-Taylor and Fehrenbach (2012) is presented in Table 6.

**Table 6.** The limits of agreement between chronological age and estimated age using four dental development charts and atlas.

Variable	*P*-value	Mean difference	Limit of agreement
The Atlas of Dental Development in the Indonesian Population	0.813	0.121 (CI: −0.069 to 0.311)	−0.969 to 1.210
The London Atlas of Human Tooth Development and Eruption by AlQahtani (2010)	0.740	−0.012 (CI: −0.361 to 0.337)	−2.013 to 1.990
The Age Estimation Guide-Modern Australia population by Blenkin-Taylor (2012)	0.571	0.051 (CI: −0.393 to 0.496)	−2.495 to 2.598
The Dental Development Chart by Ubelaker (1978)	0.675	0.165 (CI: −0.380 to 0.710)	−2.960 to 3.289

## Discussion

Tooth during development period is commonly used for age estimation. The first assessment of dental development was published by Schour and Massler in 1940 [9], based on research by Logan and Kronfeld published in 1933 [16]. Although this is the most widely used method, critics suggested that the research was based on a too small sample size of chronically ill children [17,18]. In 1978, a major revision of the assessment of Schour and Massler was made by Ubelaker, producing a chart of dental formation and eruption based on the American Indian population and relevant published literature [10,17]. In 2010, AlQahtani produced The London Atlas of Human Tooth Development and Eruption based on White and Bangladeshi ethnic groups [11]. In 2012, Blenkin and Taylor produced the Chart of Age Estimation Guide based on the modern Australian population [12]. These studies suggest that individuals of the same ethnicity had similar patterns of dental development, whereas individuals of different ethnicities showed significant variation. Since tooth development is influenced by ethnicity, variation can occur in the timing and rates of tooth development between different populations.

The assessment of dental development for the Indonesian population developed in this study aims to address the population issue. Although environmental factors such as socio-economic or nutritional status of a child influence the time of tooth eruption [19], many studies have shown that tooth development is more controlled by genes than environmental factors [8]. The rates of tooth development are influenced 90% by genetic factors. Therefore, even within the same population, there will always be some variation in dental development among individuals [12].

The samples utilized in this study ranged in age between 5 and 23 years. This age range was selected because the beginning of the mixed dentition period is around 5 years and the end of permanent dentition development and eruption is approximately 23 years. In the Indonesian population, the apical third molar is completely closed at 22 years for males and at 23–24 years for females [20]. From the legal aspect, forensic cases in Indonesia that require proof of age often occur in the age range of 8–23 years. Ages 12 and 15 years are crucial in cases of sexual misconduct. Ages 8 and 18 years are important in determining legal proceedings against children. Ages 18 and 21 years are the age limits for migrant workers. Ages 16 and 19 years are the age limits of marriage. Age 17 is the minimal age requirement to obtain a driving licence. Those ages are vulnerable to falsification. Age estimation is also important in the field of sports such as badminton and soccer. Falsification of age in soccer athletes often occurs, and the ages of 14, 16, 19, and 23 years are the age division of the national soccer team by The Football Association of Indonesia. For these reasons, the age range employed in this study was 5 to 23 years.

The dental development parameters analysed in this study were the stages of deciduous tooth root resorption, permanent tooth calcification, and permanent tooth eruption which can be observed on a panoramic radiograph, as it shows all maxillary and mandibular teeth. Since examination is visual, it is non-destructive and non-invasive methods [21]. Although panoramic radiographs have the possibility of enlargement and horizontal or vertical distortion, these limitations did not significantly affect the accuracy of this study because the assessments were qualitative [22]. In estimating age, many forensic specialists prefer to use an atlas rather than the scoring methods because it is simpler, less time-consuming, does not require specific skills in determining the stage, no calculations are needed, and only simple radiographic equipment is required [12]. Another advantage of the atlas method is that the development of every tooth can be assessed individually eliminating the need to analyse all the teeth or certain teeth as in the calculation methods.

The basic construction of the Atlas of Dental Development in the Indonesian Population consisted of analysing the modal stage of root resorption of deciduous tooth, calcification of permanent tooth, and the eruption of the permanent tooth for each tooth in each age group. This method differed from that used by AlQahtani in constructing the London Atlas of Human Tooth Development and Eruption, which used the median stage to construct the atlas [11]. There are limitations when using the median stage, as not every variable has a median stage when there is an even number of stages.

The analysis results of the dental development between males and females showed no significant differences (*P* > 0.05). However, in one case the root resorption of tooth 64 in 6-year-old age group, showed significant value 0.041 (*P* < 0.05). Although many studies suggest that there is sexual dimorphism in dental development [12], this study showed that there was very minimal dimorphism in dental development in the Indonesian population.

Analysis of dental development in this study showed that in the Indonesian population the right and left regions displayed no significant differences (*P* > 0.05). The analysis of differences in development between maxillary and mandibular teeth showed numerous significantly different variables (*P* < 0.05). The most notable difference was found in the calcification stage of the first and second incisors at the age of 5–10 years. This result was in accordance with the study of Nolla [23] that showed the differences of permanent teeth calcification stage between the maxillary and mandibular first incisorsteeth: 10–18 months for the first incisors of children aged 3.5–11 years old and 14–18 months for the second incisors of children age 4–12 years old, while the other permanent teeth calcification differences was only 0–9 months.

The Atlas of Dental Development in the Indonesian Population (Figure 4) illustrates the right region of the maxillary and mandibular teeth for combined sex. It consisted of 19 age categories of dental development from age 5 years ± 6 months to 23 years± 6 months. The ± value means that each age category is applicable to that age plus and minus 6 months of age. For example, the 5 years ±6 months age category is the modal stage of samples from age 4 years 6 months to 5 years 6 months. The atlas consisted of 17 diagrams of dental development because between age 17 years 6 months to 19 years 6 months, the modal stage of the dental development were similar, therefore age category was grouped into one diagram of 18 years 6 months ± 12 months. The similar dental development stage also occurred between 20 years 6 months to 22 years 6 month, which then grouped into one diagram of 21 years 6 months ± 12 months.

The main differences of The Atlas of Dental Development in the Indonesian Population compared to other available dental atlas methods are: 1) the sample test were from the Indonesian population, 2) the atlas was constructed by analysing the modal stage of root resorption of deciduous tooth, calcification of permanent tooth, and the eruption of the permanent tooth, 3) the age category distribution was uniform and even, and the 4) tooth calcification stages used in this atlas were the modified Demirjian et al. (1973) stages, which consisted of simpler and less stages (6 stages for anterior teeth, and 8 stages for posterior teeth).

Results demonstrate that the most accurate method applied to the Indonesian sample was the newly formed Atlas of Dental Development in the Indonesian Population (−0.969 to 1.210 years) followed by The London Atlas of Human Tooth Development and Eruption by AlQahtani (−2.013 to 1.990 years), the Age Estimation Guide-Modern Australia population by Blenkin-Taylor (−2.495 to 2.598 years) and the Dental Development Chart by Ubelaker (−2.960 to 3.289 years). These findings show that the Atlas of Dental Development constructed in this study performs better than the other three methods providing greater accuracy of age estimation in Indonesian population.

Another study [24] showed that The London Atlas of Human Tooth Development and Eruption by AlQahtani, constructed based on the research of White and Bangladeshi, estimated age more accurately and greater precision than The Dental Development Chart by Ubelaker and the chart by Schour and Massler on 1 506 test samples whose ethnic origin was Bangladeshi and white British, and only 12% from Portugal, The Netherlands, USA, Canada, and France. However, a study by Goltz [25] on 50 modern population of American-born children of Anglo-Saxon or Teutonic origins, children of Sicilian immigrants, or Black children of combined sex showed that The Dental Development Chart by Ubelaker, which was constructed based on American Indian population, was found to best estimate the age of an individual, while the chart by Schour and Massler and The London Atlas by AlQahtani overestimated the age of an individual and performed equally well.

These studies suggest that individuals of the same ancestry and ethnicity shows similar patterns of dental development, though there are genetic variations between individuals [12]. The Atlas of Dental Development in the Indonesian Population gave a more accurate result of age estimation. The other dental development methods derived from individuals of different ancestry were less suitable for Indonesian populations.

The samples for this study were mostly obtained from the Mongoloid race of Indonesian population. The limited ethnicity information of the retrospective data and the samples of intra-uterine period until 4 years age group was not included in the study. These study limitations suggest for a further prospective study with a more proportional random sampling of wider ethnic or race of Indonesian population and a more complete age range.

## Conclusion

An age estimation method using assessment deciduous tooth root resorption, permanent tooth calcification, and permanent tooth eruption in 5–23-year-old Indonesian individuals has been constructed. There was minimal sexual dimorphism and no significant differences between right and left teeth in implementing this method. However, there were significant differences in dental development between maxillary and mandibular teeth. The Atlas of Dental Development constructed in this study allowed more accurate age estimates of the Indonesian sample than the other three methods tested. Further research is required to examine the accuracy of the Atlas of Dental Development in various ethnic groups within Indonesia.

## Supplementary Material

Supplemental Material
