# Supplementary material for: Age estimation of individuals aged 5–23 years based on dental development of the Indonesian population
Source: Forensic Sci Res. 2021 Apr 15;7(2):115–23. doi: 10.1080/20961790.2021.1886648 (PMC9245992; doi:10.1080/20961790.2021.1886648)
Supplement: Supplemental Material [file TFSR_A_1886648_SM9366.docx]

**Supplementary Table S1. Modal, minimum, and maximum stage of root resorption, calcification, and eruption of the maxillary and mandibular right teeth in each age group (combined sex).**

| **Age** | **Maxillary right** | | | | | **Mandibular right** | | | | |
| --- | --- | --- | --- | --- | --- | --- | --- | --- | --- | --- |
| **5 years ± 6 months** | **Tooth** | **Number of teeth** | **Root resorption stage** | | | **Tooth** | **Number of teeth** | **Root resorption stage** | | |
|  |  |  | **Min** | **Max** | **Modal** |  |  | **Min** | **Max** | **Modal** |
|  | 55 | 16 | H | H | H | 81 | 16 | Res ½ | Exfo | Res ½ |
|  | 54 | 16 | H | Res ¼ | H | 82 | 16 | Res ¼ | Exfo | Res ½ |
|  | 53 | 16 | H | H | H | 83 | 16 | H | H | H |
|  | 52 | 16 | Res ¼ | Res ½ | Res ¼ | 84 | 16 | H | H | H |
|  | 51 | 16 | Res ¼ | Res ¾ | Res ½ | 85 | 16 | H | Res ¼ | H |
|  |  |  | **Calcification stage** | | |  |  | **Calcification stage** | | |
|  |  |  | **Min** | **Max** | **Modal** |  |  | **Min** | **Max** | **Modal** |
|  | 18 | 16 | 0 | 0 | 0 | 41 | 16 | D | F | E |
|  | 17 | 16 | B | C | C | 42 | 16 | C | E | D |
|  | 16 | 16 | D | F | E | 43 | 16 | C | E | C |
|  | 15 | 16 | B | D | C | 44 | 16 | C | D | C |
|  | 14 | 16 | C | D | C | 45 | 16 | B | D | C |
|  | 13 | 16 | C | E | D | 46 | 16 | E | F | E |
|  | 12 | 16 | C | E | C | 47 | 16 | B | C | C |
|  | 11 | 16 | C | E | D | 48 | 16 | 0 | 0 | 0 |
|  |  |  | **Eruption stage** | | |  |  | **Eruption stage** | | |
|  |  |  | **Min** | **Max** | **Modal** |  |  | **Min** | **Max** | **Modal** |
|  | 18 | 16 | 0 | 0 | 0 | 41 | 16 | Erup1 | Erup3 | Erup1 |
|  | 17 | 16 | Erup1 | Erup1 | Erup1 | 42 | 16 | Erup1 | Erup3 | Erup1 |
|  | 16 | 16 | Erup1 | Erup3 | Erup2 | 43 | 16 | Erup1 | Erup1 | Erup1 |
|  | 15 | 16 | Erup1 | Erup1 | Erup1 | 44 | 16 | Erup1 | Erup1 | Erup1 |
|  | 14 | 16 | Erup1 | Erup1 | Erup1 | 45 | 16 | Erup1 | Erup1 | Erup1 |
|  | 13 | 16 | Erup1 | Erup1 | Erup1 | 46 | 16 | Erup1 | Erup4 | Erup2 |
|  | 12 | 16 | Erup1 | Erup1 | Erup1 | 47 | 16 | Erup1 | Erup1 | Erup1 |
|  | 11 | 16 | Erup1 | Erup1 | Erup1 | 48 | 16 | 0 | 0 | 0 |
| **6 years ± 6 months** | **Tooth** | **Number of teeth** | **Root resorption stage** | | | **Tooth** | **Number of teeth** | **Root resorption stage** | | |
|  |  |  | **Min** | **Max** | **Modal** |  |  | **Min** | **Max** | **Modal** |
|  | 55 | 16 | H | Res ¼ | H | 81 | 16 | Res ½ | Exfo | Exfo |
|  | 54 | 16 | H | Res ¼ | Res ¼ | 82 | 16 | Res ¼ | Exfo | Res ½ |
|  | 53 | 16 | H | H | H | 83 | 16 | H | H | H |
|  | 52 | 16 | Res ½ | Res ½ | Res ½ | 84 | 16 | H | Res ¼ | H |
|  | 51 | 16 | Res ½ | Exfo | Res ½ | 85 | 16 | H | Res ¼ | H |
|  |  |  | **Calcification stage** | | |  |  | **Calcification stage** | | |
|  |  |  | **Min** | **Max** | **Modal** |  |  | **Min** | **Max** | **Modal** |
|  | 18 | 16 | 0 | 0 | 0 | 41 | 16 | E | F | E |
|  | 17 | 16 | B | D | C | 42 | 16 | D | F | E |
|  | 16 | 16 | E | G | E | 43 | 16 | D | E | D |
|  | 15 | 16 | C | D | D | 44 | 16 | C | E | D |
|  | 14 | 16 | C | E | D | 45 | 16 | C | F | C |
|  | 13 | 16 | C | E | D | 46 | 16 | E | G | F |
|  | 12 | 16 | C | E | D | 47 | 16 | C | D | C |
|  | 11 | 16 | D | F | E | 48 | 16 | 0 | A | 0 |
|  |  |  | **Eruption stage** | | |  |  | **Eruption stage** | | |
|  |  |  | **Min** | **Max** | **Modal** |  |  | **Min** | **Max** | **Modal** |
|  | 18 | 16 | 0 | 0 | 0 | 41 | 16 | Erup1 | Erup4 | Erup1 |
|  | 17 | 16 | Erup1 | Erup1 | Erup1 | 42 | 16 | Erup1 | Erup3 | Erup1 |
|  | 16 | 16 | Erup2 | Erup4 | Erup3 | 43 | 16 | Erup1 | Erup1 | Erup1 |
|  | 15 | 16 | Erup1 | Erup1 | Erup1 | 44 | 16 | Erup1 | Erup1 | Erup1 |
|  | 14 | 16 | Erup1 | Erup1 | Erup1 | 45 | 16 | Erup1 | Erup1 | Erup1 |
|  | 13 | 16 | Erup1 | Erup1 | Erup1 | 46 | 16 | Erup1 | Erup4 | Erup3 |
|  | 12 | 16 | Erup1 | Erup1 | Erup1 | 47 | 16 | Erup1 | Erup1 | Erup1 |
|  | 11 | 16 | Erup1 | Erup3 | Erup1 | 48 | 16 | 0 | Erup1 | 0 |
| **7 years ± 6 months** | **Tooth** | **Number of teeth** | **Root resorption stage** | | | **Tooth** | **Number of teeth** | **Root resorption stage** | | |
|  |  |  | **Min** | **Max** | **Modal** |  |  | **Min** | **Max** | **Modal** |
|  | 55 | 16 | H | Res ¼ | H | 81 | 16 | Res ¾ | Exfo | Exfo |
|  | 54 | 16 | H | Res ½ | Res ¼ | 82 | 16 | Res ¼ | Exfo | Exfo |
|  | 53 | 16 | H | H | H | 83 | 16 | H | Res ¼ | H |
|  | 52 | 16 | Res ¼ | Exfo | Res ½ | 84 | 16 | H | Res ¼ | H |
|  | 51 | 16 | Res ½ | Exfo | Res ½ | 85 | 16 | H | Res ¼ | H |
|  |  |  | **Calcification stage** | | |  |  | **Calcification stage** | | |
|  |  |  | **Min** | **Max** | **Modal** |  |  | **Min** | **Max** | **Modal** |
|  | 18 | 16 | 0 | A | 0 | 41 | 16 | E | G | F |
|  | 17 | 16 | C | D | D | 42 | 16 | D | G | F |
|  | 16 | 16 | E | G | F | 43 | 16 | C | F | E |
|  | 15 | 16 | C | D | D | 44 | 16 | C | E | E |
|  | 14 | 16 | C | E | D | 45 | 16 | C | E | D |
|  | 13 | 16 | D | E | E | 46 | 16 | E | G | G |
|  | 12 | 16 | D | F | E | 47 | 16 | C | D | D |
|  | 11 | 16 | E | G | E | 48 | 16 | 0 | A | 0 |
|  |  |  | **Eruption stage** | | |  |  | **Eruption stage** | | |
|  |  |  | **Min** | **Max** | **Modal** |  |  | **Min** | **Max** | **Modal** |
|  | 18 | 16 | 0 | Erup1 | 0 | 41 | 16 | Erup1 | Erup4 | Erup4 |
|  | 17 | 16 | Erup1 | Erup1 | Erup1 | 42 | 16 | Erup1 | Erup4 | Erup1 |
|  | 16 | 16 | Erup2 | Erup4 | Erup4 | 43 | 16 | Erup1 | Erup1 | Erup1 |
|  | 15 | 16 | Erup1 | Erup1 | Erup1 | 44 | 16 | Erup1 | Erup1 | Erup1 |
|  | 14 | 16 | Erup1 | Erup1 | Erup1 | 45 | 16 | Erup1 | Erup1 | Erup1 |
|  | 13 | 16 | Erup1 | Erup1 | Erup1 | 46 | 16 | Erup3 | Erup4 | Erup4 |
|  | 12 | 16 | Erup1 | Erup4 | Erup1 | 47 | 16 | Erup1 | Erup1 | Erup1 |
|  | 11 | 16 | Erup1 | Erup4 | Erup1 | 48 | 16 | 0 | Erup1 | 0 |
| **8 years ± 6 months** | **Tooth** | **Number of teeth** | **Root resorption stage** | | | **Tooth** | **Number of teeth** | **Root resorption stage** | | |
|  |  |  | **Min** | **Max** | **Modal** |  |  | **Min** | **Max** | **Modal** |
|  | 55 | 16 | Res ¼ | Res ½ | Res ½ | 81 | 16 | Exfo | Exfo | Exfo |
|  | 54 | 16 | Res ½ | Exfo | Res ½ | 82 | 16 | Res ¾ | Exfo | Exfo |
|  | 53 | 16 | Res ¼ | Res ½ | Res ¼ | 83 | 16 | Res ¼ | Exfo | Res ¼ |
|  | 52 | 16 | Res ½ | Exfo | Exfo | 84 | 16 | Res ¼ | Exfo | Res ½ |
|  | 51 | 16 | Res ½ | Exfo | Exfo | 85 | 16 | Res ¼ | Exfo | Res ¼ |
|  |  |  | **Calcification stage** | | |  |  | **Calcification stage** | | |
|  |  |  | **Min** | **Max** | **Modal** |  |  | **Min** | **Max** | **Modal** |
|  | 18 | 16 | 0 | A | 0 | 41 | 16 | G | H | G |
|  | 17 | 16 | D | E | D | 42 | 16 | F | H | G |
|  | 16 | 16 | F | G | G | 43 | 16 | D | F | F |
|  | 15 | 16 | D | E | E | 44 | 16 | E | F | E |
|  | 14 | 16 | D | E | E | 45 | 16 | D | E | E |
|  | 13 | 16 | D | F | E | 46 | 16 | F | G | G |
|  | 12 | 16 | D | F | F | 47 | 16 | C | E | D |
|  | 11 | 16 | D | G | F | 48 | 16 | 0 | A | 0 |
|  |  |  | **Eruption stage** | | |  |  | **Eruption stage** | | |
|  |  |  | **Min** | **Max** | **Modal** |  |  | **Min** | **Max** | **Modal** |
|  | 18 | 16 | 0 | Erup1 | 0 | 41 | 16 | Erup4 | Erup4 | Erup4 |
|  | 17 | 16 | Erup1 | Erup1 | Erup1 | 42 | 16 | Erup1 | Erup4 | Erup3 |
|  | 16 | 16 | Erup3 | Erup4 | Erup4 | 43 | 16 | Erup1 | Erup1 | Erup1 |
|  | 15 | 16 | Erup1 | Erup1 | Erup1 | 44 | 16 | Erup1 | Erup2 | Erup1 |
|  | 14 | 16 | Erup1 | Erup3 | Erup1 | 45 | 16 | Erup1 | Erup1 | Erup1 |
|  | 13 | 16 | Erup1 | Erup1 | Erup1 | 46 | 16 | Erup4 | Erup4 | Erup4 |
|  | 12 | 16 | Erup1 | Erup4 | Erup1 | 47 | 16 | Erup1 | Erup2 | Erup1 |
|  | 11 | 16 | Erup1 | Erup4 | Erup4 | 48 | 16 | 0 | Erup1 | 0 |
| **9 years ± 6 months** | **Tooth** | **Number of teeth** | **Root resorption stage** | | | **Tooth** | **Number of teeth** | **Root resorption stage** | | |
|  |  |  | **Min** | **Max** | **Modal** |  |  | **Min** | **Max** | **Modal** |
|  | 55 | 16 | Res ¼ | Exfo | Res ½ | 81 | 16 | Exfo | Exfo | Exfo |
|  | 54 | 16 | Res ½ | Exfo | Res ½ | 82 | 16 | Exfo | Exfo | Exfo |
|  | 53 | 16 | Res ¼ | Res ¾ | Res ¼ | 83 | 16 | Res ¼ | Exfo | Res ½ |
|  | 52 | 16 | Res ½ | Exfo | Exfo | 84 | 16 | Res ½ | Exfo | Res ½ |
|  | 51 | 16 | Exfo | Exfo | Exfo | 85 | 16 | Res ¼ | Exfo | Res ¼ |
|  |  |  | **Calcification stage** | | |  |  | **Calcification stage** | | |
|  |  |  | **Min** | **Max** | **Modal** |  |  | **Min** | **Max** | **Modal** |
|  | 18 | 16 | 0 | B | 0 | 41 | 16 | G | H | H |
|  | 17 | 16 | D | E | E | 42 | 16 | F | G | G |
|  | 16 | 16 | F | G | G | 43 | 16 | E | F | F |
|  | 15 | 16 | D | F | E | 44 | 16 | E | F | F |
|  | 14 | 16 | D | F | E | 45 | 16 | D | F | E |
|  | 13 | 16 | E | F | F | 46 | 16 | F | G | G |
|  | 12 | 16 | E | G | F | 47 | 16 | D | E | E |
|  | 11 | 16 | E | G | G | 48 | 16 | 0 | B | 0 |
|  |  |  | **Eruption stage** | | |  |  | **Eruption stage** | | |
|  |  |  | **Min** | **Max** | **Modal** |  |  | **Min** | **Max** | **Modal** |
|  | 18 | 16 | 0 | Erup1 | 0 | 41 | 16 | Erup4 | Erup4 | Erup4 |
|  | 17 | 16 | Erup1 | Erup2 | Erup1 | 42 | 16 | Erup3 | Erup4 | Erup4 |
|  | 16 | 16 | Erup4 | Erup4 | Erup4 | 43 | 16 | Erup1 | Erup2 | Erup1 |
|  | 15 | 16 | Erup1 | Erup3 | Erup1 | 44 | 16 | Erup1 | Erup3 | Erup1 |
|  | 14 | 16 | Erup1 | Erup3 | Erup1 | 45 | 16 | Erup1 | Erup3 | Erup1 |
|  | 13 | 16 | Erup1 | Erup1 | Erup1 | 46 | 16 | Erup4 | Erup4 | Erup4 |
|  | 12 | 16 | Erup1 | Erup4 | Erup4 | 47 | 16 | Erup1 | Erup2 | Erup1 |
|  | 11 | 16 | Erup4 | Erup4 | Erup4 | 48 | 16 | 0 | Erup1 | 0 |
| **10 years ± 6 months** | **Tooth** | **Number of teeth** | **Root resorption stage** | | | **Tooth** | **Number of teeth** | **Root resorption stage** | | |
|  |  |  | **Min** | **Max** | **Modal** |  |  | **Min** | **Max** | **Modal** |
|  | 55 | 16 | Res ½ | Exfo | Res ½ | 81 | 16 | Exfo | Exfo | Exfo |
|  | 54 | 16 | Res ½ | Exfo | Exfo | 82 | 16 | Res ½ | Exfo | Exfo |
|  | 53 | 16 | Res ¼ | Exfo | Res ½ | 83 | 16 | Res ¼ | Exfo | Exfo |
|  | 52 | 16 | Res ¾ | Exfo | Exfo | 84 | 16 | Res ½ | Exfo | Exfo |
|  | 51 | 16 | Exfo | Exfo | Exfo | 85 | 16 | Res ¼ | Exfo | Exfo |
|  |  |  | **Calcification stage** | | |  |  | **Calcification stage** | | |
|  |  |  | **Min** | **Max** | **Modal** |  |  | **Min** | **Max** | **Modal** |
|  | 18 | 16 | A | D | B | 41 | 16 | G | H | H |
|  | 17 | 16 | D | F | E | 42 | 16 | F | H | H |
|  | 16 | 16 | G | H | G | 43 | 16 | E | G | F |
|  | 15 | 16 | D | G | F | 44 | 16 | E | G | F |
|  | 14 | 16 | D | G | F | 45 | 16 | D | G | F |
|  | 13 | 16 | D | G | F | 46 | 16 | G | H | G |
|  | 12 | 16 | E | H | G | 47 | 16 | D | F | E |
|  | 11 | 16 | F | H | G | 48 | 16 | A | D | B |
|  |  |  | **Eruption stage** | | |  |  | **Eruption stage** | | |
|  |  |  | **Min** | **Max** | **Modal** |  |  | **Min** | **Max** | **Modal** |
|  | 18 | 16 | Erup1 | Erup1 | Erup1 | 41 | 16 | Erup4 | Erup4 | Erup4 |
|  | 17 | 16 | Erup1 | Erup3 | Erup1 | 42 | 16 | Erup1 | Erup4 | Erup4 |
|  | 16 | 16 | Erup4 | Erup4 | Erup4 | 43 | 16 | Erup1 | Erup4 | Erup3 |
|  | 15 | 16 | Erup1 | Erup4 | Erup1 | 44 | 16 | Erup1 | Erup4 | Erup1 |
|  | 14 | 16 | Erup1 | Erup4 | Erup1 | 45 | 16 | Erup1 | Erup4 | Erup1 |
|  | 13 | 16 | Erup1 | Erup4 | Erup1 | 46 | 16 | Erup4 | Erup4 | Erup4 |
|  | 12 | 16 | Erup1 | Erup4 | Erup4 | 47 | 16 | Erup1 | Erup4 | Erup1 |
|  | 11 | 16 | Erup3 | Erup4 | Erup4 | 48 | 16 | Erup1 | Erup1 | Erup1 |
| **11 years ± 6 months** | **Tooth** | **Number of teeth** | **Root resorption stage** | | | **Tooth** | **Number of teeth** | **Root resorption stage** | | |
|  |  |  | **Min** | **Max** | **Modal** |  |  | **Min** | **Max** | **Modal** |
|  | 55 | 16 | Res ½ | Exfo | Exfo | 81 | 16 | Exfo | Exfo | Exfo |
|  | 54 | 16 | Exfo | Exfo | Exfo | 82 | 16 | Exfo | Exfo | Exfo |
|  | 53 | 16 | Res ¾ | Exfo | Exfo | 83 | 16 | Res ½ | Exfo | Exfo |
|  | 52 | 16 | Exfo | Exfo | Exfo | 84 | 16 | Exfo | Exfo | Exfo |
|  | 51 | 16 | Exfo | Exfo | Exfo | 85 | 16 | Res ¾ | Exfo | Exfo |
|  |  |  | **Calcification stage** | | |  |  | **Calcification stage** | | |
|  |  |  | **Min** | **Max** | **Modal** |  |  | **Min** | **Max** | **Modal** |
|  | 18 | 16 | B | D | C | 41 | 16 | G | H | H |
|  | 17 | 16 | E | G | G | 42 | 16 | G | H | H |
|  | 16 | 16 | G | H | H | 43 | 16 | F | G | G |
|  | 15 | 16 | F | G | F | 44 | 16 | F | G | G |
|  | 14 | 16 | F | G | G | 45 | 16 | F | G | F |
|  | 13 | 16 | F | G | G | 46 | 16 | G | H | H |
|  | 12 | 16 | G | H | H | 47 | 16 | E | G | F |
|  | 11 | 16 | G | H | H | 48 | 16 | B | D | B |
|  |  |  | **Eruption stage** | | |  |  | **Eruption stage** | | |
|  |  |  | **Min** | **Max** | **Modal** |  |  | **Min** | **Max** | **Modal** |
|  | 18 | 16 | Erup1 | Erup1 | Erup1 | 41 | 16 | Erup4 | Erup4 | Erup4 |
|  | 17 | 16 | Erup1 | Erup4 | Erup3 | 42 | 16 | Erup4 | Erup4 | Erup4 |
|  | 16 | 16 | Erup4 | Erup4 | Erup4 | 43 | 16 | Erup1 | Erup4 | Erup4 |
|  | 15 | 16 | Erup1 | Erup4 | Erup4 | 44 | 16 | Erup3 | Erup4 | Erup4 |
|  | 14 | 16 | Erup4 | Erup4 | Erup4 | 45 | 16 | Erup1 | Erup4 | Erup4 |
|  | 13 | 16 | Erup1 | Erup4 | Erup4 | 46 | 16 | Erup4 | Erup4 | Erup4 |
|  | 12 | 16 | Erup4 | Erup4 | Erup4 | 47 | 16 | Erup2 | Erup4 | Erup4 |
|  | 11 | 16 | Erup4 | Erup4 | Erup4 | 48 | 16 | Erup1 | Erup1 | Erup1 |
| **12 years ± 6 months** | **Tooth** | **Number of teeth** | **Root resorption stage** | | | **Tooth** | **Number of teeth** | **Root resorption stage** | | |
|  |  |  | **Min** | **Max** | **Modal** |  |  | **Min** | **Max** | **Modal** |
|  | 55 | 16 | Res ½ | Exfo | Exfo | 81 | 16 | Exfo | Exfo | Exfo |
|  | 54 | 16 | Res ¾ | Exfo | Exfo | 82 | 16 | Exfo | Exfo | Exfo |
|  | 53 | 16 | Res ¼ | Exfo | Exfo | 83 | 16 | Exfo | Exfo | Exfo |
|  | 52 | 16 | Exfo | Exfo | Exfo | 84 | 16 | Res ¾ | Exfo | Exfo |
|  | 51 | 16 | Exfo | Exfo | Exfo | 85 | 16 | Res ½ | Exfo | Exfo |
|  |  |  | **Calcification stage** | | |  |  | **Calcification stage** | | |
|  |  |  | **Min** | **Max** | **Modal** |  |  | **Min** | **Max** | **Modal** |
|  | 18 | 16 | B | E | C | 41 | 16 | H | H | H |
|  | 17 | 16 | E | G | G | 42 | 16 | G | H | H |
|  | 16 | 16 | G | H | H | 43 | 16 | F | H | G |
|  | 15 | 16 | F | H | G | 44 | 16 | F | H | G |
|  | 14 | 16 | F | H | G | 45 | 16 | E | H | G |
|  | 13 | 16 | F | H | G | 46 | 16 | F | H | H |
|  | 12 | 16 | F | H | H | 47 | 16 | E | G | G |
|  | 11 | 16 | G | H | H | 48 | 16 | A | E | B |
|  |  |  | **Eruption stage** | | |  |  | **Eruption stage** | | |
|  |  |  | **Min** | **Max** | **Modal** |  |  | **Min** | **Max** | **Modal** |
|  | 18 | 16 | Erup1 | Erup1 | Erup1 | 41 | 16 | Erup4 | Erup4 | Erup4 |
|  | 17 | 16 | Erup2 | Erup4 | Erup3 | 42 | 16 | Erup3 | Erup4 | Erup4 |
|  | 16 | 16 | Erup4 | Erup4 | Erup4 | 43 | 16 | Erup2 | Erup4 | Erup4 |
|  | 15 | 16 | Erup1 | Erup4 | Erup4 | 44 | 16 | Erup1 | Erup4 | Erup4 |
|  | 14 | 16 | Erup1 | Erup4 | Erup4 | 45 | 16 | Erup1 | Erup4 | Erup4 |
|  | 13 | 16 | Erup1 | Erup4 | Erup4 | 46 | 16 | Erup4 | Erup4 | Erup4 |
|  | 12 | 16 | Erup4 | Erup4 | Erup4 | 47 | 16 | Erup2 | Erup4 | Erup4 |
|  | 11 | 16 | Erup4 | Erup4 | Erup4 | 48 | 16 | Erup1 | Erup1 | Erup1 |
| **13 years ± 6 months** | **Tooth** | **Number of teeth** | **Calcification stage** | | | **Tooth** | **Number of teeth** | **Calcification stage** | | |
|  |  |  | **Min** | **Max** | **Modal** |  |  | **Min** | **Max** | **Modal** |
|  | 18 | 16 | B | E | C | 41 | 16 | G | H | H |
|  | 17 | 16 | F | G | G | 42 | 16 | G | H | H |
|  | 16 | 16 | G | H | H | 43 | 16 | F | H | G |
|  | 15 | 16 | G | H | G | 44 | 16 | G | H | H |
|  | 14 | 16 | G | H | H | 45 | 16 | F | H | G |
|  | 13 | 16 | F | H | G | 46 | 16 | G | H | H |
|  | 12 | 16 | G | H | H | 47 | 16 | F | H | G |
|  | 11 | 16 | G | H | H | 48 | 16 | B | E | C |
|  |  |  | **Eruption stage** | | |  |  | **Eruption stage** | | |
|  |  |  | **Min** | **Max** | **Modal** |  |  | **Min** | **Max** | **Modal** |
|  | 18 | 16 | Erup1 | Erup3 | Erup1 | 41 | 16 | Erup4 | Erup4 | Erup4 |
|  | 17 | 16 | Erup2 | Erup4 | Erup4 | 42 | 16 | Erup4 | Erup4 | Erup4 |
|  | 16 | 16 | Erup4 | Erup4 | Erup4 | 43 | 16 | Erup4 | Erup4 | Erup4 |
|  | 15 | 16 | Erup4 | Erup4 | Erup4 | 44 | 16 | Erup4 | Erup4 | Erup4 |
|  | 14 | 16 | Erup4 | Erup4 | Erup4 | 45 | 16 | Erup3 | Erup4 | Erup4 |
|  | 13 | 16 | Erup3 | Erup4 | Erup4 | 46 | 16 | Erup4 | Erup4 | Erup4 |
|  | 12 | 16 | Erup3 | Erup4 | Erup4 | 47 | 16 | Erup3 | Erup4 | Erup4 |
|  | 11 | 16 | Erup4 | Erup4 | Erup4 | 48 | 16 | Erup1 | Erup3 | Erup1 |
| **14 years ± 6 months** | **Tooth** | **Number of teeth** | **Calcification stage** | | | **Tooth** | **Number of teeth** | **Calcification stage** | | |
|  |  |  | **Min** | **Max** | **Modal** |  |  | **Min** | **Max** | **Modal** |
|  | 18 | 16 | B | E | C | 41 | 16 | G | H | H |
|  | 17 | 16 | F | G | G | 42 | 16 | G | H | H |
|  | 16 | 16 | G | H | H | 43 | 16 | F | H | G |
|  | 15 | 16 | G | H | G | 44 | 16 | G | H | H |
|  | 14 | 16 | G | H | H | 45 | 16 | F | H | G |
|  | 13 | 16 | F | H | G | 46 | 16 | G | H | H |
|  | 12 | 16 | G | H | H | 47 | 16 | F | H | G |
|  | 11 | 16 | G | H | H | 48 | 16 | B | E | C |
|  |  |  | **Eruption stage** | | |  |  | **Eruption stage** | | |
|  |  |  | **Min** | **Max** | **Modal** |  |  | **Min** | **Max** | **Modal** |
|  | 18 | 16 | Erup1 | Erup2 | Erup1 | 41 | 16 | Erup4 | Erup4 | Erup4 |
|  | 17 | 16 | Erup3 | Erup4 | Erup4 | 42 | 16 | Erup4 | Erup4 | Erup4 |
|  | 16 | 16 | Erup4 | Erup4 | Erup4 | 43 | 16 | Erup4 | Erup4 | Erup4 |
|  | 15 | 16 | Erup4 | Erup4 | Erup4 | 44 | 16 | Erup4 | Erup4 | Erup4 |
|  | 14 | 16 | Erup4 | Erup4 | Erup4 | 45 | 16 | Erup4 | Erup4 | Erup4 |
|  | 13 | 16 | Erup3 | Erup4 | Erup4 | 46 | 16 | Erup4 | Erup4 | Erup4 |
|  | 12 | 16 | Erup4 | Erup4 | Erup4 | 47 | 16 | Erup3 | Erup4 | Erup4 |
|  | 11 | 16 | Erup4 | Erup4 | Erup4 | 48 | 16 | Erup1 | Erup3 | Erup1 |
| **15 years ± 6 months** | **Tooth** | **Number of teeth** | **Calcification stage** | | | **Tooth** | **Number of teeth** | **Calcification stage** | | |
|  |  |  | **Min** | **Max** | **Modal** |  |  | **Min** | **Max** | **Modal** |
|  | 18 | 16 | D | F | D | 48 | 16 | D | F | D |
|  | 17 | 16 | G | H | G | 47 | 16 | G | H | G |
|  |  |  | **Eruption stage** | | |  |  | **Eruption stage** | | |
|  |  |  | **Min** | **Max** | **Modal** |  |  | **Min** | **Max** | **Modal** |
|  | 18 | 16 | Erup1 | Erup3 | Erup1 | 48 | 16 | Erup1 | Erup4 | Erup1 |
|  | 17 | 16 | Erup3 | Erup4 | Erup4 | 47 | 16 | Erup3 | Erup4 | Erup4 |
| **16 years ± 6 months** | **Tooth** | **Number of teeth** | **Calcification stage** | | | **Tooth** | **Number of teeth** | **Calcification stage** | | |
|  |  |  | **Min** | **Max** | **Modal** |  |  | **Min** | **Max** | **Modal** |
|  | 18 | 16 | C | F | E | 48 | 16 | C | G | E |
|  | 17 | 16 | G | H | G | 47 | 16 | G | H | G |
|  |  |  | **Eruption stage** | | |  |  | **Eruption stage** | | |
|  |  |  | **Min** | **Max** | **Modal** |  |  | **Min** | **Max** | **Modal** |
|  | 18 | 16 | Erup1 | Erup3 | Erup1 | 48 | 16 | Erup1 | Erup4 | Erup1 |
|  | 17 | 16 | Erup3 | Erup4 | Erup4 | 47 | 16 | Erup3 | Erup4 | Erup4 |
| **17 years ± 6 months** | **Tooth** | **Number of teeth** | **Calcification stage** | | | **Tooth** | **Number of teeth** | **Calcification stage** | | |
|  |  |  | **Min** | **Max** | **Modal** |  |  | **Min** | **Max** | **Modal** |
|  | 18 | 16 | D | G | G | 48 | 16 | E | G | G |
|  | 17 | 16 | G | H | H | 47 | 16 | G | H | H |
|  |  |  | **Eruption stage** | | |  |  | **Eruption stage** | | |
|  |  |  | **Min** | **Max** | **Modal** |  |  | **Min** | **Max** | **Modal** |
|  | 18 | 16 | Erup1 | Erup4 | Erup1 | 48 | 16 | Erup1 | Erup4 | Erup2 |
|  | 17 | 16 | Erup4 | Erup4 | Erup4 | 47 | 16 | Erup4 | Erup4 | Erup4 |
| **18 years ± 6 months** | **Tooth** | **Number of teeth** | **Calcification stage** | | | **Tooth** | **Number of teeth** | **Calcification stage** | | |
|  |  |  | **Min** | **Max** | **Modal** |  |  | **Min** | **Max** | **Modal** |
|  | 18 | 16 | E | G | G | 48 | 16 | E | G | G |
|  | 17 | 16 | H | H | H | 47 | 16 | G | H | H |
|  |  |  | **Eruption stage** | | |  |  | **Eruption stage** | | |
|  |  |  | **Min** | **Max** | **Modal** |  |  | **Min** | **Max** | **Modal** |
|  | 18 | 16 | Erup1 | Erup4 | Erup3 | 48 | 16 | Erup1 | Erup4 | Erup3 |
|  | 17 | 16 | Erup4 | Erup4 | Erup4 | 47 | 16 | Erup4 | Erup4 | Erup4 |
| **19 years ± 6 months** | **Tooth** | **Number of teeth** | **Calcification stage** | | | **Tooth** | **Number of teeth** | **Calcification stage** | | |
|  |  |  | **Min** | **Max** | **Modal** |  |  | **Min** | **Max** | **Modal** |
|  | 18 | 16 | E | G | G | 48 | 16 | E | G | G |
|  | 17 | 16 | G | H | H | 47 | 16 | G | H | H |
|  |  |  | **Eruption stage** | | |  |  | **Eruption stage** | | |
|  |  |  | **Min** | **Max** | **Modal** |  |  | **Min** | **Max** | **Modal** |
|  | 18 | 16 | Erup1 | Erup4 | Erup3 | 48 | 16 | Erup2 | Erup4 | Erup3 |
|  | 17 | 16 | Erup4 | Erup4 | Erup4 | 47 | 16 | Erup4 | Erup4 | Erup4 |
| **20 years ± 6 months** | **Tooth** | **Number of teeth** | **Calcification stage** | | | **Tooth** | **Number of teeth** | **Calcification stage** | | |
|  |  |  | **Min** | **Max** | **Modal** |  |  | **Min** | **Max** | **Modal** |
|  | 18 | 16 | F | H | G | 48 | 16 | H | G | G |
|  | 17 | 16 | G | H | H | 47 | 16 | G | H | H |
|  |  |  | **Eruption stage** | | |  |  | **Eruption stage** | | |
|  |  |  | **Min** | **Max** | **Modal** |  |  | **Min** | **Max** | **Modal** |
|  | 18 | 16 | Erup2 | Erup4 | Erup3 | 48 | 16 | Erup2 | Erup4 | Erup4 |
|  | 17 | 16 | Erup4 | Erup4 | Erup4 | 47 | 16 | Erup4 | Erup4 | Erup4 |
| **21 years ± 6 months** | **Tooth** | **Number of teeth** | **Calcification stage** | | | **Tooth** | **Number of teeth** | **Calcification stage** | | |
|  |  |  | **Min** | **Max** | **Modal** |  |  | **Min** | **Max** | **Modal** |
|  | 18 | 16 | F | H | G | 48 | 16 | G | G | G |
|  | 17 | 16 | H | H | H | 47 | 16 | G | H | H |
|  |  |  | **Eruption stage** | | |  |  | **Eruption stage** | | |
|  |  |  | **Min** | **Max** | **Modal** |  |  | **Min** | **Max** | **Modal** |
|  | 18 | 16 | Erup3 | Erup4 | Erup4 | 48 | 16 | Erup3 | Erup4 | Erup4 |
|  | 17 | 16 | Erup4 | Erup4 | Erup4 | 47 | 16 | Erup4 | Erup4 | Erup4 |
| **22 years ± 6 months** | **Tooth** | **Number of teeth** | **Calcification stage** | | | **Tooth** | **Number of teeth** | **Calcification stage** | | |
|  |  |  | **Min** | **Max** | **Modal** |  |  | **Min** | **Max** | **Modal** |
|  | 18 | 16 | G | H | G | 48 | 16 | G | G | G |
|  | 17 | 16 | H | H | H | 47 | 16 | G | H | H |
|  |  |  | **Eruption stage** | | |  |  | **Eruption stage** | | |
|  |  |  | **Min** | **Max** | **Modal** |  |  | **Min** | **Max** | **Modal** |
|  | 18 | 16 | Erup3 | Erup4 | Erup4 | 48 | 16 | Erup3 | Erup4 | Erup4 |
|  | 17 | 16 | Erup4 | Erup4 | Erup4 | 47 | 16 | Erup4 | Erup4 | Erup4 |
| **23 years ± 6 months** | **Tooth** | **Number of teeth** | **Calcification stage** | | | **Tooth** | **Number of teeth** | **Calcification stage** | | |
|  |  |  | **Min** | **Max** | **Modal** |  |  | **Min** | **Max** | **Modal** |
|  | 18 | 16 | G | H | H | 48 | 16 | G | H | G |
|  | 17 | 16 | H | H | H | 47 | 16 | H | H | H |
|  |  |  | **Eruption stage** | | |  |  | **Eruption stage** | | |
|  |  |  | **Min** | **Max** | **Modal** |  |  | **Min** | **Max** | **Mode** |
|  | 18 | 16 | Erup3 | Erup4 | Erup4 | 48 | 16 | Erup3 | Erup4 | Erup4 |
|  | 17 | 16 | Erup4 | Erup4 | Erup4 | 47 | 16 | Erup4 | Erup4 | Erup4 |

Min: minimum; Max: maximum. Stages abbreviation is described in Figures 1–3.
